# Characterisation of Microbial Community Associated with Different Disinfection Treatments in Hospital hot Water Networks

**DOI:** 10.3390/ijerph17062158

**Published:** 2020-03-24

**Authors:** Stefania Paduano, Isabella Marchesi, Maria Elisabetta Casali, Federica Valeriani, Giuseppina Frezza, Elena Vecchi, Luca Sircana, Vincenzo Romano Spica, Paola Borella, Annalisa Bargellini

**Affiliations:** 1 Department of Biomedical, Metabolic and Neural Sciences, Section of Public Health, University of Modena and Reggio Emilia, 41125 Modena, Italy; stefania.paduano@unimore.it (S.P.); isabella.marchesi@unimore.it (I.M.); casali.mariaelisabetta@gmail.com (M.E.C.); giuseppina.frezza@unimore.it (G.F.); paola.borella@unimore.it (P.B.); 2 University Hospital Policlinico of Modena, 41124 Modena, Italy; vecchi.elena@aou.mo.it (E.V.); sircana.luca@aou.mo.it (L.S.); 3 Department of Movement, Human and Health Sciences, Public Health Unit, University of Rome ‘Foro Italico’, 00135 Rome, Italy; federica.valeriani@uniroma4.it (F.V.); vincenzo.romanospica@uniroma4.it (V.R.S.)

**Keywords:** environmental microbiome, waterborne opportunistic pathogens, biocides, water distribution systems, microbial ecology, 16S amplicon sequencing, hospital building

## Abstract

Many disinfection treatments can be adopted for controlling opportunistic pathogens in hospital water networks in order to reduce infection risk for immunocompromised patients. Each method has limits and strengths and it could determine modifications on bacterial community. The aim of our investigation was to study under real-life conditions the microbial community associated with different chemical (monochloramine, hydrogen peroxide, chlorine dioxide) and non-chemical (hyperthermia) treatments, continuously applied since many years in four hot water networks of the same hospital. Municipal cold water, untreated secondary, and treated hot water were analysed for microbiome characterization by 16S amplicon sequencing. Cold waters had a common microbial profile at genera level. The hot water bacterial profiles differed according to treatment. Our results confirm the effectiveness of disinfection strategies in our hospital for controlling potential pathogens such as *Legionella*, as the investigated genera containing opportunistic pathogens were absent or had relative abundances ≤1%, except for non-tuberculous mycobacteria, *Sphingomonas*, *Ochrobactrum* and *Brevundimonas*. Monitoring the microbial complexity of healthcare water networks through 16S amplicon sequencing is an innovative and effective approach useful for Public Health purpose in order to verify possible modifications of microbiota associated with disinfection treatments.

## 1. Introduction

The complexity of healthcare facilities and the increasing proportion of immunologically vulnerable patients make prevention of healthcare-associated waterborne infections a priority. Several factors make hospital buildings suitable for colonization with bacteria and moulds: large and complex water systems with areas of low flow predispose to stagnation and biofilm formation and water temperatures optimal for healthcare use may also be ideal for bacterial growth [1]. Moreover, water characteristics, age and corrosion of the pipes, or metabolic activity of colonizing bacteria can influence the microbial community [2,3,4]. Therefore, the microbial ecology of water networks varies in the water distribution systems [5] and can serve as reservoirs for waterborne pathogens such as *Legionella* spp., *Pseudomonas aeruginosa*, *Stenotrophomonas maltophilia*, *Acinetobacter* spp., and non-tuberculous mycobacteria [6,7,8,9,10,11].

A range of on-site physical and chemical treatments for secondary disinfection have been proposed with the aim of controlling microbial water contamination, differing in effectiveness, application methods (in continuous and shock), costs and management. Each of these methods has benefits and disadvantages and to date, the most effective procedure has not been defined [12,13,14]. Among physical methods, circulating water at 60 °C, such that the temperature at each outlet reaches at least 50 °C and preferably 55 °C, is the method most commonly used to control legionellae in hot water system. It is relatively easy to implement and to monitor continuously, but it has the possible disadvantage of increasing energy consumption and the risk of scalding [15]. Moreover, the temperature control regimen may fail, especially in large complex water systems with poor engineering control, leading to the necessary implementation of additional control methods, such as biocides [13,16]. Among chemical disinfection treatments, the most popular are continuous systems such chlorine-based biocides and hydrogen peroxide [17]. All these methods have proven effective against *Legionella* and other waterborne pathogens, but active concentrations of biocide need to be continuously monitored since no one eliminates the bacteria once the water network is contaminated [12,13,18,19]. The main disadvantages associated with chemical treatments are corrosiveness and, limited to chlorine-based biocides, formation of toxic disinfection by-products (DBPs) [3,12,13,20].

Wang et al. [4] suggested that disinfection treatments can modify the composition of microbial community of drinking water distribution systems both in terms of abundance and diversity at the genus level. Additionally, the effectiveness of disinfection in removing pathogens from drinking water is mediated by the microbial ecology of drinking water system [5]. For instance, the growth of nitrifying bacteria in chloraminated building plumbing systems can contribute to accelerated chloramine residuals decay [21]. Moreover, it is widely accepted that bacteria like *Legionella* spp. and *Mycobacterium* spp. embedded in biofilm are more resistant to disinfection than individual planktonic cells [22,23].

It is difficult to cultivate most environmental microorganisms under laboratory conditions, thus limiting our knowledge of the whole bacterial community [24,25]. In the last decade, the use of next generation sequencing (NGS) and bioinformatics tools has offered a more extensive approach for examining the culturable and unculturable microorganisms, allowing to characterize the entire microbial community in an ecological niche [26,27,28,29,30].

To date, there is still a notable lack of studies concerning the effect of disinfection on the microbial community present in the healthcare water networks where secondary disinfection is frequently adopted in order to reduce the risk of waterborne infections. Since drinking water distribution systems are designed and maintained to transport chemically and biologically safe potable water to consumers [31] and treatments can determine important modification in the microbiota structure present in the water networks [29], the aim of our investigation was to study under real-life conditions the microbial community associated with different chemical (monochloramine, hydrogen peroxide, chlorine dioxide) and non-chemical (hyperthermia) treatments, continuously applied since many years in four hot water networks of the same hospital, by using 16S amplicon sequencing approach.

## 2. Materials and Methods

### 2.1. Water Distribution Systems

The study was carried out in the 621-bedded University Hospital Policlinico of Modena, Italy, consisting of separate buildings constructed between 1970s and 1990s. The incoming cold groundwater, disinfected with chlorine dioxide, is provided by the municipality. A single main cold water line branches out into several supply lines, each of which enters the water station of each hospital building. Once inside, cold water is heated using heat exchangers in order to produce hot water which is moved in a water recirculation system. No secondary disinfection treatment and no storage are applied on the cold water. Continuous hydrogen peroxide, monochloramine and chlorine dioxide systems are used since many years as secondary disinfection systems on hot water lines of three different buildings defined as building 1, 2, and 3, respectively. Hydrogen peroxide device is operating since 2012, monochloramine and chlorine dioxide devices since 2009 [13]. A 48% hydrogen peroxide solution (O_2_ s.r.l., Bergamo, Italy) is injected continuously into the recirculating hot water by a dosing pump in order to ensure concentrations of 15 to 20 mg/L at distal outlets. Monochloramine is generated in situ by reaction between a stabilized chlorine-based precursor and an ammonium salt (Sanipur s.r.l., Brescia, Italy). The monochloramine generator is set to keep a concentration of biocide in the recirculation loop between 1.5 and 3.0 mg/L. Monochloramine residual levels are in line with the guideline value of 3 mg/L and the maximum contaminant level of 4 mg/L, established by WHO and the U.S. Environmental Protection Agency, respectively. Chlorine dioxide is produced in situ by injecting hydrochloric acid and sodium chlorite (Sanipur s.r.l., Brescia, Italy). A dosing system is set up to add the disinfectant to the recirculating hot water, ensuring concentrations of at least 0.3 mg/L at distal points. This dose was reported as effective in controlling *Legionella* contamination in European hospitals [32].

Hot water in a fourth building (building 4) is not chemically treated but is produced at a temperature of at least 60 °C and distributed continuously at >50 °C (hyperthermia). This hot water network supplies medical studies and laboratories, while the hospital rooms have instantaneous water heaters which are directly fed with cold water and maintained at 60 °C, for *Legionella* contamination control.

### 2.2. Samples Collection

One inlet cold water and six hot water samples were collected from every building. The cold water sample was collected within the water station at the entry of each building, while hot water was taken from different sites throughout each building (hot water heater, recirculation loop and taps). In detail, hot water samples were collected from the water network treated with hydrogen peroxide (HP) within the building 1, from the network treated with monochloramine (M) within the building 2, from the network treated with chlorine dioxide (CD) within the building 3, and from the not chemically treated network (NT) within the building 4.

Water samples were collected during two sampling sessions (February and May 2018). In February, the hot water samples were collected in duplicate (called “a” and “b”) in order to verify reproducibility of methodology. Due to the positive results, there were no replicates in May.

Two litres of hot water and five litres of inlet cold water were collected in sterile plastic bottles without flaming and after 1 min flushing. Sodium thiosulphate was added in order to neutralize residual free chlorine or other oxidizing biocides. Water temperature and residual disinfectant concentration were determined during samples collection as explained: temperature (digital thermometer), free chlorine and chlorine dioxide (DPD method, Microquant, Merck, Darmstadt, Germany), monochloramine (DR900 colorimeter, indophenol method, Hach Lange, Milan, Italy), hydrogen peroxide (reflectometer RQflex 2, Merck, Darmstadt, Germany). Collection bottles were returned to the laboratory immediately after sampling.

The University Hospital gave approval and permission to collect water samples for this study.

### 2.3. Characterization of Microbial Community

Water samples were concentrated by membrane filtration (0.40 μm polycarbonate membrane, extraction DNA kit, Minerva Biolabs, Germany). The filter membrane was stored in a Petri plate at −20 °C. The filter membrane was turned upside down onto an incubation dish filled with 2 mL of Lysis buffer (Minerva BioLabs, Germany) and then incubated at 37 °C for 30 min. Subsequently, the lysis solution was transferred into an incubation tube with 0.1 mg of glass beads (Sigma Aldrich, USA) and incubated at 56 °C for 15 min after vortexing for 1 min. DNA purification was carried out using Aqua screen Fast Extract Kit according to the manufacturer’s protocol (Minerva BioLabs, Germany).

#### Bacterial Community 16S Profiling

16S rRNA paired-end sequencing was performed according to the “16S Metagenomic Sequencing Library Preparation” protocol (Part# 15044223 rev. A; Illumina, USA). Briefly, the primers containing Illumina adapter and linker sequence and targeting the V1-V2 regions of bacterial 16S rRNA genes were used [30,33,34]. Three libraries with unique tags were generated for each sample as technical replicates. Each amplification reaction had a total volume of 25 μL containing 12.5 μL of KAPA HiFi HotStart ReadyMix (Roche, Pleasanton, CA, USA), 5 μL of each primer (1 μM), and 2 μL template DNA. Reactions were carried out on a Techne^®^TC-PLUS thermalcycler (VWR International, LLC, Radnor, USA). Following amplification, 5 μL of PCR product from each reaction was used for agarose gel (1%) electrophoresis to confirm amplification. The final concentration of cleaned DNA amplicon was determined using the Qubit PicoGreen dsDNA BR assay kit (Invitrogen, Grand Island, NY, USA) and validated on Bioanalyzer DNA 1000 chip (Agilent, Santa Clara, CA, USA).

Due to failed DNA amplification, 10 out of 80 samples collected were excluded. Failed DNA amplification may have been due to multiple factors: low DNA concentrations, the presence of PCR inhibitors (i.e., humic substances, phenolic compounds) and/or extensive DNA damage caused by high levels of disinfectant (i.e., chlorine) [31,35,36].

Libraries were prepared using the MiSeq Reagent Kit Preparation Guide (Illumina, San Diego, CA, USA). Raw sequence data was processed using an in-house pipeline which was built on the Galaxy platform and incorporated various software tools to evaluate the quality of the raw sequence data (e.g., FastQC, http://www.bioinformatics.babraham.ac.uk/projects/fastqc/). All data sets were rigorously screened to remove low quality reads (short reads <200 nt, zero-ambiguous sequences). Demultiplexing was performed to remove PhiX sequences and sort sequences; moreover, to minimize sequencing errors and ensure sequence quality, the reads were trimmed based on the sequence quality score using Btrim [37]. OTUs (operational taxonomic units) were clustered at a 97% similarity level and final OTUs were generated based on the clustering results. Taxonomic annotation of individual OTUs was based on representative sequences using RDP’s 16S Classifier 2.5. Observed OTUs were defined as observed species. The sequence reads were analysed, also, in the cloud environment BaseSpace through the 16S Metagenomics app (version 1.0.1 (Illumina^®^, San Diego, CA, USA)): the taxonomic database used was the Illumina-curated version (May 2013 release of the Greengenes Consortium Database [38]).

The raw sequencing data have been submitted to NCBI Sequence Read Archive (http://www.ncbi.nlm.nih.gov/sra/) with the project accession number of PRJNA574025.

### 2.4. Data Analysis

Relative abundances of community members were determined with rarefied data and summarized at each taxonomic level. Alpha and Beta diversity were calculated using EstimateS software at a level of 97% sequence similarity. Regarding Alpha diversity, microbial richness was computed based on number of OTUs observed and biodiversity was estimated through Shannon index and equitability index at species level [39]. Morisita Horn index (Beta diversity) was calculated in order to evaluate similarity/dissimilarity between two samples or two pools [40,41,42]. The index ranges from 0 (no similarity) to 1 (complete similarity). Principal components analysis (PCA) was performed using METAGENassist platform [43], in order to investigate the dissimilarity between groups. Moreover, Beta diversity clustering was analysed using ANOSIM (ANalysis Of SIMilarities) and it was run to assess significant differences in relative abundance of OTUs on different samples [44]. In order to assess sequencing depth, alpha rarefaction plots were done in mothur (version 1.31.1, www.mothur.org) and R (version 3.1.3, www.R-project.org) using packages ‘ggplot2’ and ‘vegan’ (R Core team 2013).

All statistical analyses were performed with software package IBM SPSS statistics, version 22 (IBM Corp., Armonk, NY, USA) Comparison for normal variables was performed by *t*-test and one-way analysis of variance (ANOVA) with the Bonferroni test and for non-normal variables (OTUs) by non-parametric test (Mann–Whitney test and Kruskal–Wallis test). The Pearson correlation coefficient and the Spearman’s rank correlation coefficient were calculated to analyse the correlation between normal variables and between non-normal variables, respectively.

## 3. Results

A total of 70 samples were analysed (8 cold water and 62 hot water samples). Forty-six samples (65.7%) were collected in February during the first sampling session and the remaining in May (second session). Rarefaction curves were calculated for each sample (Figure S1). All the curves reached a stable plateau. Separately amplified and barcoded replicates of hot water samples from the first sampling session (*n*. 19 couples) were sequenced to verify reproducibility of procedure. Morisita Horn analyses of replicates demonstrated high levels of community similarity at genera level, with an average equal to 0.904 ± 0.130 and values higher than 0.850 for 89% of replicates (*n*. 17 couples) and higher than 0.540 for the remaining 11% of the replicates (*n*. 2 couples).

### 3.1. Cold Water Samples

**Figure 1:**
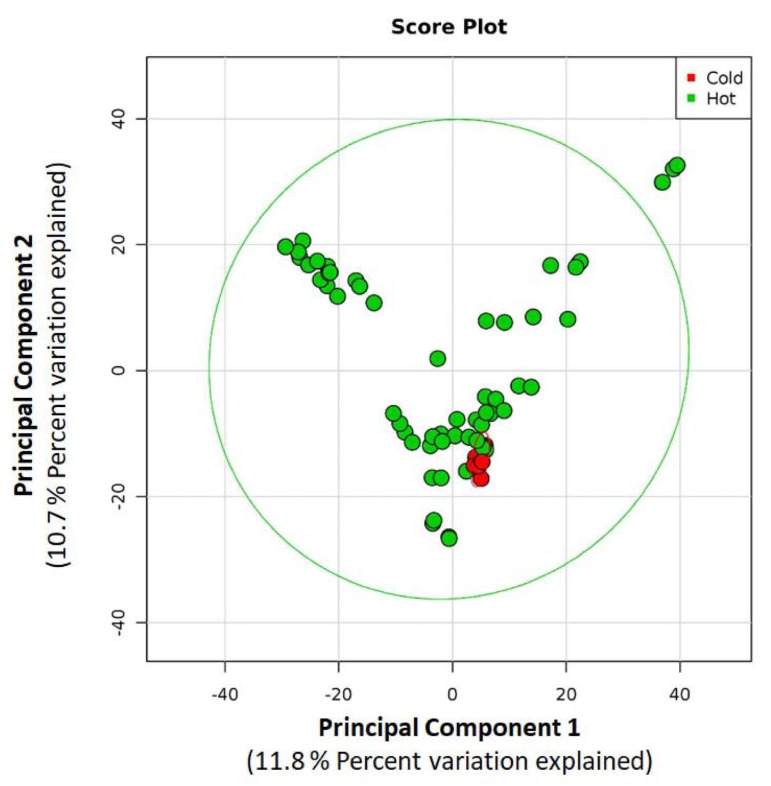
Principal components analysis (PCA) of the normalized relative abundance of 16S rRNA sequences in all samples. Data are plotted following the genus-level classification. The PCA demonstrates the clustering of 16S rRNA sequences from cold water (Red) and hot water (Green).

**Figure 2:**
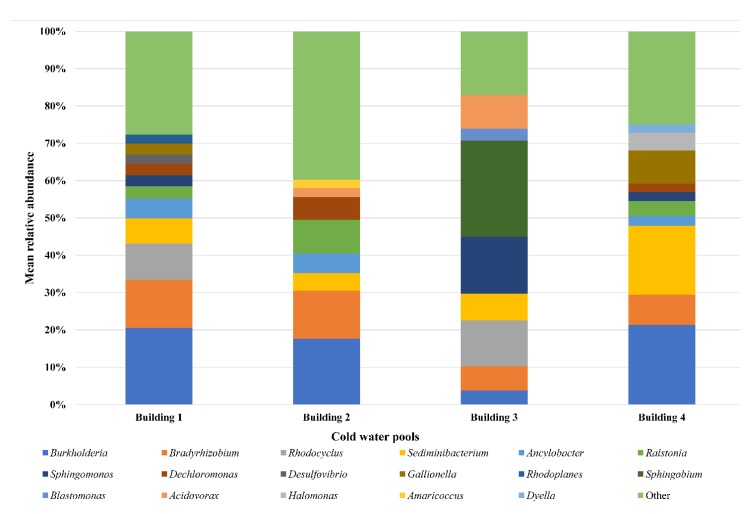
Distribution of genera in inlet cold water pools according to building (cut off 2%). Genera with relative abundance ≤ 2% are listed as “other”.

Eight cold water samples were analysed, 4 from the first and 4 from the second sampling session. Table 1 shows the values of temperature, OTUs, Shannon and equitability indices for each sample. No significant differences were observed for waters collected in February compared to those collected in May. The Principal Components Analysis showed common profiles for all the cold waters (Figure 1). The similarity of cold waters from four different buildings was supported by ANOSIM (R value = 0.208, *p* = 0.112). In cold water samples, the predominant *phyla* were Proteobacteria (mean 76.30%), Bacteroidetes (mean 11.50%) and Actinobacteria (mean 7.75%). Figure 2 shows the predominant genera in inlet cold water to the four buildings. *Burkholderia* (mean 15.82%), *Bradyrhizobium* (mean 10.11%) and *Sediminibacterium* (mean 9.24%) were the predominant and common genera for all cold water samples.

### 3.2. Hot Water Samples

A total of 62 hot water samples were analysed, 14 treated with hydrogen peroxide (HP), 17 with monochloramine (M), 15 with chlorine dioxide (CD), and 16 not chemically treated (NT). The mean temperature of the samples collected during the first sampling session (*n*. 42) did not differed from the mean of those collected during the second one (*n*. 20) (46.0 ± 5.6 vs 43.2 ± 7.0 °C).

Table 2 presents water temperatures, biocide concentrations, Shannon and equitability indices and number of OTUs. Samples from network NT had significantly higher mean temperature than the other networks (HP, M, CD). Kruskal–Wallis test showed a global statistically significant difference in terms of OTUs (*p* = 0.008). Median values of OTUs resulted significantly higher in not chemically treated network than in networks treated with hydrogen peroxide and monochloramine (*p* = 0.001 NT vs HP; *p* = 0.038 NT vs M; *p* = 0.286 NT vs CD, Mann–Whitney test), while no significant differences in OTUs median were detected between the three chemically treated hot networks (*p* = 0.115, Kruskal–Wallis test). No significant differences were observed between the four hot networks in terms of alpha diversity expressed as Shannon (*p* = 0.481) and Equitability (*p* = 0.254) indices (ANOVA). Also limiting the analysis to the three networks chemically treated, ANOVA tests showed no significant differences in terms of Shannon index (*p* = 0.385) and Equitability index (*p* = 0.228). Moreover, we observed a negative, although not significant, correlation between the number of OTUs and biocide concentration, with Spearman’s Rho equal to −0.225 (*p* = 0.440) for hydrogen peroxide, −0.153 (*p* = 0.557) for monochloramine and −0.318 (*p* = 0.248) for chlorine dioxide.

**Figure 3:**
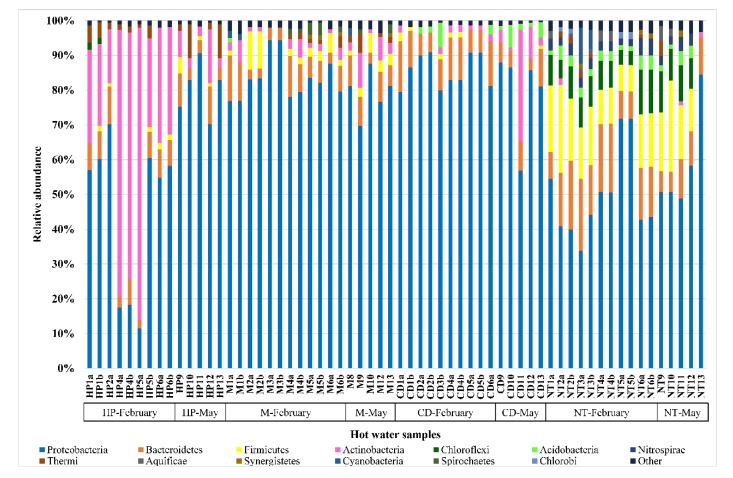
Distribution of *phyla* in hot water samples (cut off 1%). *Phyla* with relative abundance ≤1% are included in "other". HP: network with hydrogen peroxide, M: network with monochloramine, CD: network with chlorine dioxide, and NT: not chemically treated network.

Figure 3 shows the distribution of the relative abundance of *phyla* for each hot water sample. The predominant *phylum* was Proteobacteria (69.31%), followed by Actinobacteria (8.83%), Bacteroidetes (8.77%), Firmicutes (5.22%), Thermi (1.37%), and Acidobacteria (1.36%). The mean relative abundance of Proteobacteria was higher in the samples from network M (82.13%) and network CD (83.58%) compared to samples from network HP (57.85%) or network NT (52.34%). Conversely, the *phylum* Actinobacteria was prevalent in the samples from network HP (29.87%) compared to those from network M (3.03%) or network CD (4.28%) or network NT (0.89%). The *phyla* Bacteroidetes and Firmicutes had a higher mean relative abundance in not chemically treated hot water samples (12.87% and 14.52%, respectively) compared to the chemically treated ones. The mean relative abundance of Thermi was higher in the samples from network HP (3.23%) compared to those from other networks. Relative abundance of Acidobacteria was higher in network CD (2.37%) and network NT (2.55%) than other networks.

**Figure 4:**
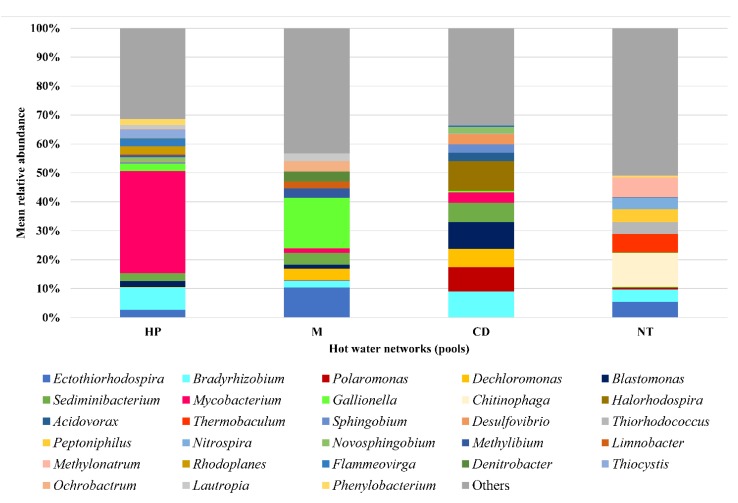
Distribution of genera in hot water samples (cut off 2%), clustering for hot water network (pools). Genera with relative abundance ≤2% are included in "other". HP: network with hydrogen peroxide, M: network with monochloramine, CD: network with chlorine dioxide, and NT: not chemically treated network.

**Figure 5:**
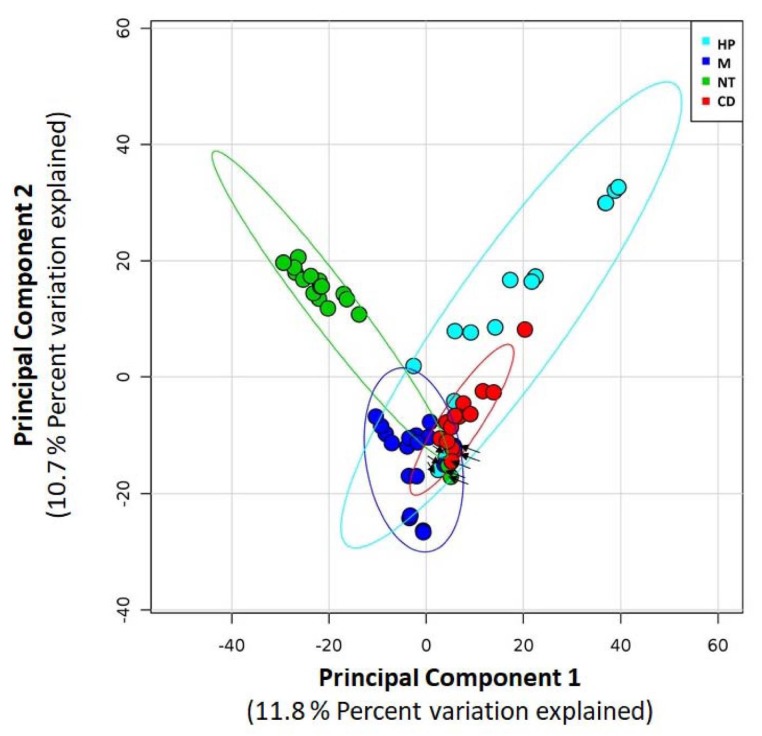
Principal components analysis (PCA) of the normalized relative abundance of 16S rRNA sequences in all samples. Data are plotted following the genus-level classification. Light blue dots for network with hydrogen peroxide (HP), blue for monochloramine (M), red for chlorine dioxide (CD), and green for not chemically treated (NT). Black arrows indicate inlet cold water samples for each network.

The genera distribution of samples pooled for the four hot water networks is illustrated in Figure 4 and the three predominant genera for each network are shown in Table 3. Some genera resistant to unfavourable environmental conditions were predominant in the presence of biocides, such as *Bradyrhizobium* (networks treated with hydrogen peroxide and chlorine dioxide), *Mycobacterium* (hydrogen peroxide), *Gallionella* (monochloramine) and *Blastomonas* (chlorine dioxide). The thermophilic genera (*Thermobaculum*) predominated in network NT. The Morisita Horn indices were calculated by comparing the four pools in order to evaluate the similarity/dissimilarity of microbiological community. This analysis revealed different microbiological profiles at genera level according to the water networks with the average value of the Morisita Horn indices equal to 0.221 (range 0.118−0.294). The microbiological similarity of the hot water samples from the same network emerged from the Principal Components Analysis, as shown in Figure 5. Moreover, ANOSIM was calculated by comparing the four hot waters pools. The result indicated a higher dissimilarity between hot waters from different networks than those within the same network (R value = 0.621, *p* = 0.001).

In networks HP and CD there was no association between the biocide concentration and genera relative abundances, while *Sediminibacterium* negatively correlated with the monochloramine concentration in network M (Pearson = −0.580, *p* = 0.015).

### 3.3. Genera Containing Opportunistic Pathogens

Sequence data was further analysed in order to observe the differences in genera containing opportunistic pathogens among the different hot water networks. *Escherichia, Shigella, Salmonella, Klebsiella* and *Enterobacter* genera were investigated but not detected in any samples. Genera detected were *Burkholderia, Brevundimonas, Sphingomonas, Mycobacterium, Legionella, Acinetobacter, Chryseobacterium, Ochrobactrum, Pseudomonas,* and *Stenotrophomonas*. All these genera had relative abundances ≤1%, except for *Mycobacterium* in the chemically treated networks, *Sphingomonas* in all networks, *Ochrobactrum* in network M (mean 4.03%) and *Brevundimonas* in network CD (mean 1.07%). *Mycobacterium* had higher relative abundance in hot water samples treated with hydrogen peroxide (mean 36.36%) followed by chlorine dioxide (4.16%) and monochloramine (1.80%), and lower levels in NT hot water network (mean 0.07%). Mean relative abundance of *Sphingomonas* was <2% in all networks (range 1.05%–1.71%).

We also investigated species for each genus containing opportunistic pathogen with mean relative abundance above 1% in hot water (Table S1). We identified 46 species of non-tuberculous *Mycobacterium*, 28 species of *Sphingomonas,* none of which associated with human infection, 6 species of *Ochrobactrum*, among which *O. anthropi*, and 5 species of *Brevundimonas,* included *B. diminuta*.

## 4. Discussion

In this investigation, we evaluated under real-life conditions the microbial community associated with different chemical and non-chemical (hyperthermia) treatments, by using 16S amplicon sequencing approach. We analysed both cold and hot waters collected in four buildings of our University hospital, all receiving the same incoming cold water but differing in secondary disinfection treatments continuously applied to hot water since many years in order to reduce the risk of waterborne infections, as reported in previous papers [13,17,45].

Similar to other studies [31,46], the inlet cold water provided by the municipality presented a common profile in all hospital buildings, characterized by the same predominant *phyla* and the same predominant environmental ubiquitous genera. The first three predominant *phyla* in hot water were the same found in cold water samples, which are common constituents of water microbial community [4,47]. Interestingly, hot water networks showed a different microbiological profile at genera level in relation to treatment. In not chemically treated water network, heat-resistant genera were among the predominant, confirming that temperature influences the water microbial community [48,49]. Indeed, the water temperatures of this network were above 50 °C compared to chemically treated ones, which were kept at ≤45 °C in order to avoid the degradation of biocides. In the chemically treated networks, the genera resistant to unfavourable environmental conditions were predominant. *Bradyrhizobium* was mostly present in the networks treated with hydrogen peroxide and chlorine dioxide. *Mycobacterium*, *Gallionella* and *Blastomonas* were found among the prevalent genera in network treated with hydrogen peroxide, monochloramine and chlorine dioxide, respectively.

*Bradyrhizobium* belongs to the Rhizobiaceae family [50]. Rhizobiaceae includes nitrogen-fixing bacteria producing nitrogenase enzyme, which reduces molecular nitrogen (N2) in ammonia (NH3). Of note, our results showed a lower relative abundance of *Bradyrhizobium* in water network treated with monochloramine than the other chemically treated ones, in accordance with Gomez-Alvarez et al. [51]. The authors studied the biofilm community succession in bench-scale annular reactors simulating arrestment of chloraminated drinking water nitrification. In more detail, *Bradyrhizobium* dominated in early stages of biofilm formation when reduced nutrient levels found in water habitats together with minimal monochloramine residuals (<0.1 mg/l) provided the metabolically versatile *Bradyrhizobium* a competitive advantage to outcompete other biofilm groups. In last periods of biofilm formation, the increase of monochloramine residual from ≈0.4 to 3.0 mg/l led to a decline in *Bradyrhizobium* relative abundance.

*Mycobacterium* genus is ubiquitous in the environment, especially in water [4,46,52]. This genus had higher relative abundance in the chemically treated networks compared to the not chemically treated one, in line with other investigations [46,53,54]. These studies reported higher *Mycobacterium* relative abundances in the samples treated with monochloramine, while in our hospital the highest relative abundance (36.4%) have been achieved in network treated with hydrogen peroxide. The dissimilarity between chemically and not chemically treated hot waters highlights *Mycobacterium* resistance to biocides, which eliminate or reduce the bacterial genera sensitive to their action, allowing the proliferation of the resistant bacteria. Regarding the possible survival mechanisms, the self-produced extracellular polymeric substances (EPS) are responsible for surface adhesion and allow the biofilm formation [55], that can protect *Mycobacterium* from direct exposure to disinfectants [56]. Although the composition of EPS varies significantly among different bacterial species, exopolysaccharides, proteins and lipids remain the key components of EPS [57]. The mycobacterial pellicles contain large quantities of free mycolic acids [58,59], that contribute to increase the cell-surface hydrophobicity to facilitate the cell-to-cell interaction for biofilm formation. Biofilm is difficult to penetrate by the disinfectants; thus, bacteria are protected inside it. Since monochloramine is able to penetrate biofilm [23], this mechanism can explain the dissimilarity of *Mycobacterium* relative abundance between hydrogen peroxide and monochloramine treated networks in our hospital.

In network with monochloramine treatment, *Gallionella* was the predominant genus. It is an iron oxidizing bacterium, aerobic microorganism that derive energy from the oxidation of iron from the ferrous state (Fe^2+^) to the ferric state (Fe^3+^) by accelerating the natural reaction between oxygen and ferrous ions in the water or on the surface of metal pipes such as galvanized iron, cast iron and steel pipes [60,61,62]. The presence of *Gallionella* has a potential negative effect on the pipes, due to the precipitation of its metabolism product (hydrated ferric hydroxide) reducing the pipes section [60]. Members of Gallionellaceae family requires also ammonia in the environment for autotrophic growth, as reported by other authors [61,63]. A previous study reported high *Gallionella* concentration in groundwater containing high levels of iron, manganese and ammonia [64]. Moreover, Stanish et al. reported that seasonally chloramine-treated systems had significantly higher abundances of several metal-oxidizing taxa including *Gallionella* spp. than chlorinated systems [65]. Since the incomplete reaction of monochloramine precursors can lead the ammonia release [66], it is not surprising that *Gallionella* was the predominant genus in waters treated with this biocide. This information can be relevant in monitoring of this disinfection treatment. In case of increased ammonia concentrations, corrective actions have to be taken in order to ensure that the proportions of chlorine and ammonia precursors during monochloramine formation are correct.

Regarding the *Blastomonas* genus, the relative abundance was higher in hot water treated with chlorine dioxide, suggesting a resistance to this treatment. Other studies found the *Blastomonas* resistance to chlorination [67,68]. Soto-Giron et al. detected *Blastomonas* as the second most abundant genus, after *Mycobacterium*, in hospital shower hoses [69]. They have also reported that *Blastomonas* and *Mycobacterium* expressed genes associated with resistance to disinfectants, including genes encoding proteins, such as catalase and superoxide dismutase, able to confer protection against oxidative stress [69,70].

A lower microbial richness (OTUs number) was observed in chemically treated hot water than in not-chemically treated ones. It is possible that disinfectants, killing some selected vulnerable genera, allowed an increase of dominance of the disinfectant-resistant microbial groups, thereby decreasing OTUs counts. Indeed, the negative, although not significant, correlation between the OTUs number and each biocide concentration suggests a decrease number of bacterial species with the increasing disinfectant concentration. A recent study about monochloramine impact on the microbiota of a hospital water network confirmed a lower bacterial richness in treated samples compared to control [71].

The overall microbial community composition was similar within each water network during our study as confirmed by PCA analysis, thus suggesting that the observed differences may be influenced by the treatment. The selective pressure of disinfectants on the microbial community has been observed also in other studies [4,54,72,73].

Several waterborne pathogen-containing genera were examined in order to observe the differences in relative abundance due to the disinfectant in use. The concentrations of biocides were at levels effective in reducing *Legionella* contamination [17,74]. Our results confirmed the effectiveness of biocides used in our hospital for controlling the presence of potential pathogens such as *Legionella*, as most of the investigated genera containing opportunistic pathogens were absent or present at very low level of relative abundance (≤1%), even if it cannot be excluded that low levels of pathogenic bacteria may pose a potential risk to public health. The increased relative abundance of non-tuberculous mycobacteria, especially in waters treated with hydrogen peroxide underlines that there exists the potential for unwanted consequences of supplemental disinfectant addition. Understanding the impact of secondary disinfection on water system microbial community is necessary to maximize biocide effectiveness and to ensure that supplemental disinfectant does not select for alternative opportunistic pathogens [53], also considering that other system factors such as temperature and pipe materials may impact microbial ecology [4]. In addition, although the 16S amplicon sequencing method has the limitation of not providing information about live/dead status, it can help identify these “hot spots” that could be acted upon with culture-based methods which are likely to remain the gold standard. In a context of risk prevention and rapid screening, molecular methods allow to support more extensive, timely, and economical evaluation of problematic sites that could be followed-up upon with culturing [75].

A limitation of this study is the absence of a pre-post treatment approach, not applicable as the programme to control *Legionella* contamination in the hospital’s hot water distribution systems was implemented since 2000 in accordance with mandatory National Guidelines. At that time, the hospital buildings were highly contaminated and over years the most promising disinfection treatments have been selected to better control the contamination, as reported in a previous paper [14]. In addition, other variables would be needed to better understand building-to-building variations, but this study aims to be one of the first approaches under real-life conditions. Most of the prior research in this area investigated bench or pilot-scale simulated water distribution systems [4,31,51,54], where a rigorous control of numerous variables such as pipe materials, degree of stagnation, average ambient temperature is possible. However, important strengths of our study are to investigate simultaneously microbiota of four different hospital hot water networks and to look at the incoming cold water for microbial profile as well as longitudinally at the system over different seasons in order to observe how this affects microbial communities. The high level of similarity among replicates is an evidence of reproducibility of our procedure.

Our research provides novel knowledge about the influence of disinfection on microbial community within water distribution systems under real-life conditions, an issue still scarcely investigated that requires further studies to better comprehend the treatments’ selective pressure on microbiota of water networks in healthcare settings. As Wang et al. [76] stated, research is needed to better understand how engineering controls may individually or in combination select a desirable microbiome, and how the microbiome itself may mediate proliferation of opportunistic pathogens, suggesting a probiotic approach to pathogens control in premise plumbing system.

## 5. Conclusions

We found different microbial profiles associated with four hot water networks. These findings may be related to the selective pressures exerted by disinfection systems on microbial communities. Monitoring the microbial complexity of healthcare water networks through 16S amplicon sequencing is an innovative and effective approach in its application in Public Health in order to verify possible modifications of microbiota associated with disinfection.

## Figures and Tables

**Table 1:** Temperature (t), operational taxonomic units (OTUs), Shannon and equitability index for each cold water sample.

Inlet Cold Water					
Month	Sample	t (°C)	OTUs	Shannon Index	Equitability Index
February	Building 1	13.0	518	1.967	0.340
	Building 2	12.9	664	2.305	0.327
	Building 3	12.9	350	1.772	0.363
	Building 4	12.8	484	1.503	0.344
		mean	median	mean	mean
		12.9	501	1.887	0.344
May	Building 1	13.8	291	2.527	0.267
	Building 2	13.4	383	2.507	0.332
	Building 3	12.5	280	1.919	0.366
	Building 4	16.7	376	2.062	0.393
		mean	median	mean	mean
		14.1	334	2.254	0.340

**Table 2:** Comparison between hot water according to the networks. HP: network with hydrogen peroxide, M: network with monochloramine, CD: network with chlorine dioxide, and NT: not chemically treated network.

Hot Water Parameters	Network HP	Network M	Network CD	Network NT	*p*-Value
Temperature Mean (°C) (Range)	45.2 (33.4–49.2)	40.0 (31.0–46.3)	42.1 (36.2–44.9)	53.2 (50.4–56.6)	<0.0001
Biocide concentration Mean (ppm) (Range)	21.3 (18.1–25.0)	3.0 (2.6–3.6)	0.2 (0.1–0.4)	-	-
OTUs Median (Range)	341 (274–414)	384 (252–816)	410 (271–956)	509 (312–981)	0.008
Shannon index Mean (Range)	2.316 (0.874–3.549)	2.110 (1.488–2.674)	1.998 (0.926–2.769)	2.135 (1.544–2.532)	0.481
Equitability index Mean (Range)	0.395 (0.155–0.629)	0.353 (0.249–0.433)	0.327 (0.165–0.487)	0.343 (0.259–0.425)	0.254

**Table 3:** The three predominant genera for each hot water network. HP: network with hydrogen peroxide, M: network with monochloramine, CD: network with chlorine dioxide and NT: not chemically treated network.

Network	Predominant Genera	Relative Abundance (%)		
		Mean	Minimum	Maximum
HP	*Mycobacterium*	36.36	1.59	84.50
	*Bradyrhizobium*	8.58	2.23	22.77
	*Thiocystis*	3.45	0.43	11.10
M	*Gallionella*	17.71	0.96	60.42
	*Ectothiorhodospira*	10.42	0.44	27.11
	*Sediminibacterium*	4.24	1.45	9.87
CD	*Halorhodospira*	10.35	0.06	51.93
	*Blastomonas*	9.72	1.48	37.28
	*Bradyrhizobium*	8.91	3.37	25.82
NT	*Chitinophaga*	11.70	4.04	21.06
	*Methylonatrum*	6.90	0.02	50.20
	*Thermobaculum*	6.19	2.42	12.30

## Supplementary Materials

The following are available online at https://www.mdpi.com/1660-4601/17/6/2158/s1. Figure S1: Rarefaction curves of the bacterial 16S rRNA gene sequences from the 70 water samples based on OTUs calculated at 97% identity. The total number of sequences analysed is plotted against the number of OTUs observed in the same community. For hot water samples: HP: network with hydrogen peroxide, M: network with monochloramine, CD: network with chlorine dioxide and NT: not chemically treated network. For cold water samples: CW: Cold Water, Building is indicated as 1, 2, 3 and 4, F: February and M: May, Table S1: Identified species of investigated genera containing opportunistic pathogens in hot waters.
