# Supplementary material for: Characterisation of Microbial Community Associated with Different Disinfection Treatments in Hospital hot Water Networks
Source: Int J Environ Res Public Health. 2020 Mar 24;17(6):2158. doi: 10.3390/ijerph17062158 (PMC7143765; doi:10.3390/ijerph17062158)
Supplement: Supplementary file 1 [file ijerph-17-02158-s001.zip › Supplementary materials/S1 Table.docx]

**S1 Table. Identified species of investigated genera containing opportunistic pathogens in hot waters.**

| *Mycobacterium*  (46 species) | *Sphyngomonas*  (28 species) | *Ochrobactrum*  (6 species) | *Brevundimonas*  (5 species) |
| --- | --- | --- | --- |
| *heckeshornense*  *sydneyiensis*  *gordonae*  *hackensackense*  *heidelbergense*  *seoulense*  *buckleii*  *frederiksbergense*  *vanbaalenii*  *neoaurum*  *montefiorense*  *savoniae*  *cosmeticum*  *kuopiense*  *arupense*  *caprae*  *alsiensis*  *lentiflavum*  *diernhoferi*  *shinjukuense*  *aichiense*  *vaccae*  *abscessus*  *heraklionense*  *hassiacum*  *lepromatosis*  *duvalii*  *pinnipedii*  *scrofulaceum*  *interjectum*  *pyrenivorans*  *psychrotolerans*  *isoniacini*  *pallens*  *kubicae*  *neglectum*  *novocastrense*  *shottsii*  *salmoniphilum*  *gilvum*  *senuense*  *smegmatis*  *hiberniae*  *chitae*  *tilburgii*  *acapulcensis* | *sanxanigenens*  *oligophenolica*  *leidyia*  *mathurensis*  *ginsenosidimutans*  *desiccabilis*  *asaccharolytica*  *wittichii*  *suberifaciens*  *japonica*  *elodea*  *hunanensis*  *soli*  *mali*  *roseiflava*  *panni*  *abaci*  *yabuuchiae*  *echinoides*  *hankookensis*  *phyllosphaerae*  *melonis*  *kwangyangensis*  *insulae*  *sanguinis*  *dokdonensis*  *parapaucimobilis*  *azotifigens* | *thiophenivorans*  *anthropi*  *pseudogrignonense*  *rhizosphaerae*  *pecoris*  *lupini* | *diminuta*  *olei*  *terrae*  *staleyi*  *bullata* |
