# Supplementary figures and images for: Characterisation of Microbial Community Associated with Different Disinfection Treatments in Hospital hot Water Networks
Source: Int J Environ Res Public Health. 2020 Mar 24;17(6):2158. doi: 10.3390/ijerph17062158 (PMC7143765; doi:10.3390/ijerph17062158)

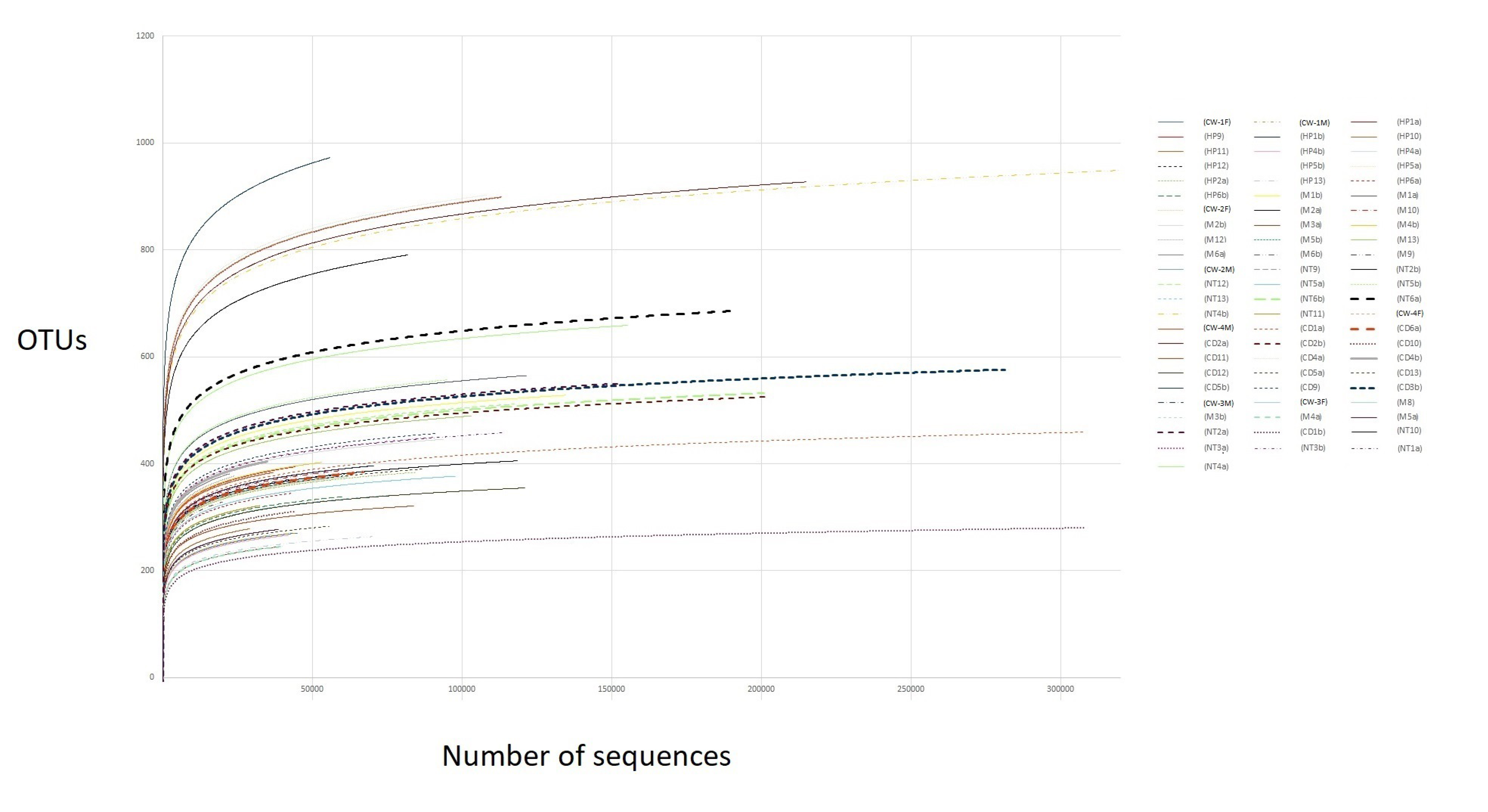

Supplement: Supplementary file 1 [file ijerph-17-02158-s001.zip › Supplementary materials/S1 Fig.tif]
